# Effects of Long-Term Repeated Freeze-Thaw Cycles on the Engineering Properties of Compound Solidified/Stabilized Pb-Contaminated Soil: Deterioration Characteristics and Mechanisms

**DOI:** 10.3390/ijerph17051798

**Published:** 2020-03-10

**Authors:** Zhongping Yang, Xuyong Li, Denghua Li, Yao Wang, Xinrong Liu

**Affiliations:** 1 College of Civil Engineering, Chongqing University, Chongqing 400045, China; 201916021050@cqu.edu.cn (X.L.); Ldhua_better@163.com (D.L.); a_yao0218@163.com (Y.W.); liuxrong@126.com (X.L.); 2 Key Laboratory of New Technology for Construction of Cities in Mountain Area (Chongqing University), Ministry of Education, Chongqing 400045, China; 3 National Joint Engineering Research Center for Prevention and Control of Environmental Geological Hazards in the TGR Area Chongqing University, Chongqing 400045, China; 4 China Coal Technology & Engineering Group (CCTEG) Chongqing Engineering Co., Ltd. Yuzhong District, Chongqing 400045, China

**Keywords:** heavy metal, long-term freeze-thaw cycle, solidification/stabilization, engineering properties, microscopic mechanism

## Abstract

The effects of long-term repeated freeze-thaw cycles and pollution levels on the engineering properties (*q_u_, E_50_, φ, c*, and *k*) of Pb-contaminated soils were investigated in various laboratory tests. These soils were solidified/stabilized (S/S) with three types of cement-based combined binders (C2.5S5F5, C5S2.5F2.5, and C5S5, cement, lime, and fly ash, mixed in different proportions; these materials are widely used in S/S technology). The strength and permeability coefficient of compound solidified/stabilized Pb-contaminated soils (Pb-CSCSs) were determined based on measurements of unconfined compressive strength (UCS), direct shear, and permeability. CT scanning, scanning electron microscopy (SEM), and Fourier transform infrared spectroscopy (FTIR) tests were employed to analyse the deterioration mechanisms under various repetitions of freeze-thaw cycles. The results showed that, under repeated freeze-thaw cycles, the engineering properties of Pb-CSCSs all degraded to varying degrees, though degradation tended to stabilise after 30 days of freeze-thaw cycles. The study also found that the pollutants obstruct hydration and other favourable reactions within the soil structure (such as ion exchanges and agglomerations and pozzolanic reactions). The activation of hydration reactions and the rearrangement of soil particles by freeze-thaw cycles thus caused the engineering properties to fluctuate, and soils exhibited different deterioration characteristics with changes in Pb^2+^ content.

## 1. Introduction

With the rapid development of urbanisation and industrialisation in China and the strengthening of awareness of the need for environmental protection over the last few decades, many industrial manufacturing enterprises with excessive pollution risks have been shut down or relocated as part of the process of upgrading industrial structures and the adjustment of urban layouts [1]. Several studies [2,3,4] have reported that numerous abandoned industrial sites have thus been created in China, and approximately 65% of all cities in China suffer from severe or extreme heavy metal pollution in urban soils. In addition, the use of chemical fertilisers, sewage irrigation, and the discharge of solid, liquid, and gas wastes from industries all cause heavy metal pollution (Cd, Hg, As, Pb, Cr, Zn, Cu, etc.), which is estimated to reduce grain production by more than 10 million tons each year, leading to an economic loss of more than RMB 20 billion annually [5]. This means that heavy metal pollution has become the most prominent soil pollution issue in China [6].

Such heavy metal pollutants in the soil do great harm to the ecosystem and to human bodies, with particularly negative effects on the health of children [7,8,9,10], under the action of groundwater migration, acid rain, and urban sewage leaching [11]. In addition, the presence of high levels of heavy metal contaminants can significantly alter the basic physical and mechanical properties of the soil, with observed instances including increased porosity and compressibility and reduced shear strength and bearing capacity [12,13,14], many of which may cause construction safety issues. In most cases, however, soil is only treated after the occurrence of geotechnical quality incidents and there is little in the way of preventive treatment [15].

Managing heavy metal contaminated soil has attracted attention from scholars all over the world, and several promising treatment methods [16,17,18,19,20,21,22,23,24] have been investigated to reduce the harm such pollution causes. Solidification/stabilization (S/S) is among these technologies, and this has rapidly become the preferred technology for the treatment of heavy metal contaminated soil due to its short cycle, low cost, simplicity of operation, wide scope, and prevention of biodegradation. Additionally, some recent studies [25,26,27] have shown that after solidifying/stabilizing, the contaminated soil can be used as an environmentally friendly construction foundation filling material or as a building block for structures due to its high strength.

The main mechanisms used immobilize pollutants in the soil using the S/S technology are physical adsorption, chemical precipitation, displacement reactions, and complexation [28,29]. Researchers have found that the effects of these physical and chemical processes are closely related to the external environment, such as freeze-thaw cycles [30,31,32], curing temperature [33,34], acid rain infiltration [35,36,37], dry-wet cycles [38,39,40], and even the carbonation [41,42], however. Among these influences, repeated freeze-thaw processes enhance the organic mineralisation of the soil and its adsorption and desorption of organic matter [43], affecting the microbial activity and storage of free energy in soil [44,45]. The most intuitive is that the freeze-thaw cycle causes morphological changes and migration of water in the soil, which leads to fracturing of the soil structure, which can cause serious deformation of fill soil in construction [46,47,48]. The resulting changes in the physical and chemical properties of soil therefore greatly influence its engineering properties [23,31,47,49,50]. The frozen areas of China are widely distributed, with seasonally frozen soil regions and transient permafrost regions accounting for about 53.5% and 23.9% of land mass, respectively [51]. This makes it clear that the freeze-thaw cycle has a serious impact on China’s engineering construction. Furthermore, some studies [52,53,54] have reported that the performance of S/S heavy metal contaminated soil is time-dependent, yet ensuring the long-term effectiveness of S/S technology is critical to issues such as the design strength of solidified contaminated soil and the leaching risk of contaminants [55]. Such discussions of long-term effectiveness can also be used to verify the effectiveness of the repair work and provide guarantees that engineering construction work will be sufficiently safety throughout its use [56]. The longevity of the solidified contaminated soils under freezing and thawing conditions is thus particularly important, as soil engineering properties have been identified as being greatly changed under these circumstances.

However, although there have been several studies investigating the effects of freeze-thaw cycles on the engineering properties of S/S heavy metal contaminated soil, little of this has focused on their long-term performance under repeated freeze-thaw processes. Moreover, most research has focused on the unconfined compressive strength and permeability coefficient of solidified soils, while few studies have investigated changes to other important engineering properties such as the deformation modulus (*E_50_*), internal friction angle (*φ*), and cohesion (*c*) under the action of freeze-thaw cycles. Unconfined compressive strength (*q_u_*) is an index that directly reflects the bearing capacity of the soil, while permeability (*k*) is one of the main mechanical properties of soil, affecting the stresses in the soil, and thus the strength and deformation of the soil. Additionally, for S/S heavy metal contaminated soil, it also affects the leaching risk of stabilised pollutants. In addition to strength control, settlement control is often required when designing foundations, and the deformation modulus (*E_50_*) is commonly used to evaluate the settlement of foundations. Internal friction angle (*φ*) and cohesion (*c*), in contrast, are indicators of shear strength, used when calculating the bearing capacity or evaluating the stability of the foundation, and calculating the pressure on the back soil of the retaining wall. The effects of long-term freeze-thaw cycles and heavy metal contamination pose severe challenges to the safety of engineering constructions, making it urgent for further studies to investigate the effects of long-term freeze-thaw processes on the engineering properties of S/S remediated heavy metal contaminated soils.

In the authors’ previous study [57], due to the wide distribution of Pb-contaminated soil in China [6] and the use of several common binders (cement, lime, and fly ash), they systematically investigated the effect of binder dosage on the engineering properties of solidified/stabilized lead-contaminated soils, remediated with cement/lime/fly ash alone or with mixtures of these in certain proportions, under freeze-thaw cycles. It was speculated that all three kinds of cement-based compound binders, C2.5S5F5, C5S2.5F2.5, and C5S5, could simultaneously improve the unconfined compressive strength (*q_u_*), deformation modulus (*E_50_*), internal friction angle (*φ*), and cohesion (*c*) of compound solidified/stabilized Pb-contaminated soils (Pb-CSCSs) under freeze-thaw cycles, while simultaneously decreasing the permeability coefficient (*k*). This paper thus uses a high and low temperature alternating test box to simulate the alternation change process of temperature in seasonally frozen regions, with low liquid limit clay, lead nitrate, and the three types of cement-based compound binders mentioned above used to prepare artificial Pb-CSCSs. A series of laboratory experiments were then conducted to examine these materials under the effects of long-term freeze-thaw cycles (90d) to:

1. verify the resistance of such Pb-CSCSs to freeze-thaw degradation,
2. investigate the impact of Pb^2+^ content on the performance of Pb-CSCSs,
3. study the deterioration characteristics of the five engineering properties, and
4. probe into the micro-mechanisms of any changes in properties by using CT scanning, scanning electron microscopy (SEM), and Fourier transform infrared spectrum analysis (FTIR) tests.

## 2. Materials and Methods

### 2.1. Materials

The soil utilised in this test was collected from an industrial site in Chongqing, China. The soil samples were air-dried naturally in the laboratory for about two weeks, after which they were tapped lightly using a wooden mallet and sieved through a 20-mesh polyethylene sieve (<0.8 mm) to remove any stones, coarse materials, and other debris. Subsequently, liquid-plastic limit combined measurement, compaction tests, and chemical analysis were conducted to identify the soil’s physical and chemical properties (Table 1). Based on the standards for engineering classification of soil (GB/T 50145-2007) [58], the soil used in this study is classified as low liquid limit clay (CL).

Portland cement (OPC325), lime and fly ash purchased from Chongqing, China, were selected as binders for compound remediation, and their main component, as obtained through X-ray fluorescence testing, are shown in Table 2. In view of the high solubility (high cation activity) of nitrate ions, as well as their lack of interference with the remediation process, lead nitrate (P_b_(NO_3_)_2_, a chemical analytical agent) was used as a heavy metal contaminant [28]. Deionized water was used to avoid the influence of excess ions.

### 2.2. Methods

#### 2.1.1. Experimental Design

Some studies [60,61] have suggested that the ratio of pollutant content has a certain effect on the engineering properties of solidified heavy metal contaminated soil. Therefore, the three compound binder ratios (C2.5S5F5, C5S2.5F2.5, and C5S5) were used with varying content of Pb^2+^ (0.05%, 0.5%, and 1%, mass ratios of Pb^2+^ to dry soils or Pb^2+^ concentration values were 500mg/kg, 5000mg/kg, and 10,000 mg/kg, respectively) were used as variables to explore the deterioration characteristics in engineering properties of Pb-CSCSs under long-term freeze-thaw cycles (0d, 3d, 7d, 14d, 30d, and 90d). Nonparametric hypothesis testing was carried out in IBM SPSS to further explore the significance of Pb^2+^ content on the long-term effectiveness of Pb-CSCSs. Unrepaired Pb-contaminated soils with Pb^2+^ content of 1% were used as control samples.

The microstructure of soil is the underlying source of its macroscopic behaviours. Although the macroscopic mechanical properties of soil can be obtained by laboratory testing, studies of the microstructure of soil are thus also needed to understand the essence of these mechanical properties. In this work, computed tomography testing (CT), scanning electron microscopy (SEM) and Fourier transform infrared spectroscopy testing (FTIR) were conducted to explore the microscopic mechanisms of engineering properties deterioration characteristics in Pb-CSCSs after long-term freeze-thaw processes. The experiment scheme is shown in Table 3.

#### 2.1.2. Specimens Preparation

**Figure 1:**
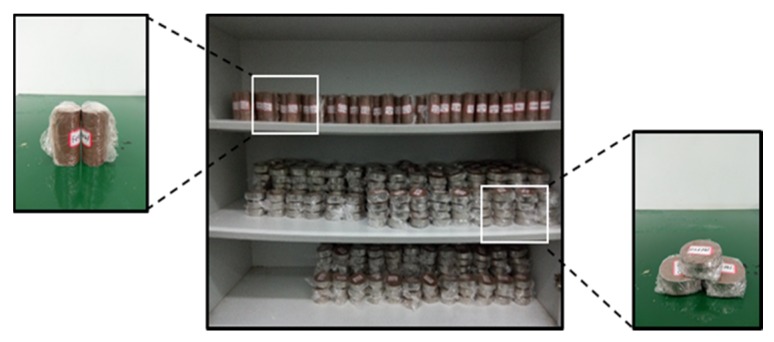
Specimens.

To obtain specimens for unconfined compressive strength tests (diameter 39.1 mm, height 80 mm), sheer strength tests (diameter 61.8 mm, height 20 mm), and permeability coefficient tests (diameter 61.8 mm, height 20 mm) (Figure 1), a predetermined mass of lead nitrate (Pb(NO_3_)_2_) was added to deionized water based on the required contaminant content level; the solution was then thoroughly mixed with a magnetic stirrer (HSC-19T, JOANLAB) at 1500 rpm for 15 min. Subsequently, the contaminant solution was added to the prepared dry soil according to the experimental scheme. The solution and soils were each mixed thoroughly with the stirrer and then packed in a plastic hermetic bag and cured for 24 h in a standard curing room to ensure that solution and dry soil were evenly mixed. The prepared contaminant soil samples were then mixed evenly with a predetermined mass of binders. To prevent moisture from evaporating, the mixed soil samples were immediately statically compacted into specimens using cylindrical stainless steel moulds at 95% of the maximum dry density of the soil samples (1.75 g/cm3). All samples were then placed in a standard curing room (20 ± 3°C, relative humidity 100%) for 28 days.

#### 2.1.3. Apparatus and Testing Methods

**Figure 2:**
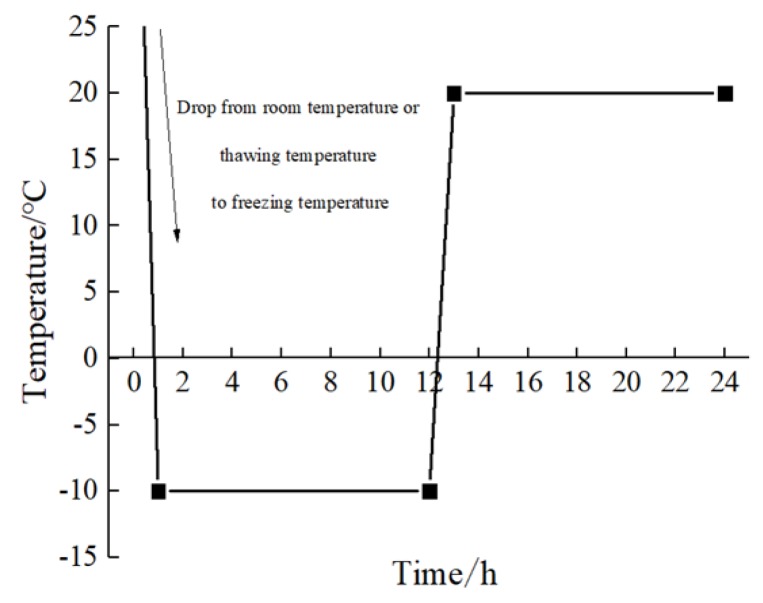
Temperature change curve of each freeze-thaw cycle.

Specimens were air-dried under natural conditions and put into the High and Low-Temperature Alternating Test Box (TC401, Chongqing Taisite Test Instrument Co. Ltd., Chongqing, China, with temperature range: −4~100 °C) for freezing and thawing after the curing process. To allow specimens to be frozen and thawed completely, each freeze-thaw cycle consisted of a constant temperature of −10 °C for 11 h followed by a constant temperature of 20 °C for 11 h. Thus, one freeze-thaw cycle was completed in one day (Figure 2).

In accordance with standards for geotechnical testing method (GB/T 50123-2019) [62], unconfined compressive strength (UCS, *qu*) was determined using a strain-controlled unconfined pressure meter (YYW-2, Nanjing Ningxi Soil Instrument Co., Ltd.) with a strain rate of 1%/min; the deformation modulus (*E_50_*) is the ratio of stress to strain (secant modulus) when the stress sustained by the sample is half (50%) of its peak stress. Shear strength (τ, rapid shear) was measured using a ZJ strain-controlled direct shear device (Nanjing soil instrument factory Co., Ltd.) with a shearing speed of 0.8 mm/min. The internal friction angle (*φ*) and cohesion (*c*) were obtained using Mohr-Coulomb theory, and the permeability coefficient (*k*) was tested using an automatic environmental geotechnical penetration meter (GDSPERM, GDS Instruments Ltd., Hook, UK). After 24 h of vacuum saturation, the sample was subjected to varying confining pressures so that the seepage pressure was constantly changed. The permeability coefficient at 20 °C water temperature was obtained from the relationship between the permeation rate of the clay soil and the hydraulic gradient [63].

The SOMATOM Scope CT scanning system (Shanghai Siemens Medical Devices Co., Ltd., with image reconstruction parameters: layer thickness 0.6–10 mm, reconstructed field of view 5–50 cm, and maximum IRS reconstruction speed 20 images/sec) was used for CT testing. As CT imaging of soil material is affected by the material of the scanning bed, the specimens were placed on a carton and the crossed red lines were aligned in the circular cavity of the scanning frame prior to the start of scanning. Three-dimensional perspectives of each sample were then formed by using Avizo and ImageJ to process the files obtained from the CT tests.

SEM experiments were carried out with a tungsten lamp scanning electron microscope (VEGA3, TESCAN CHINA, Ltd.) operating at 10kV. In order to gain better representation of the analysis area, the samples for the SEM test (20 × 20 × 30 mm) were selected from areas with relatively uniform particle and pore distributions according to the 3D perspectives, which were then dried in a dryer at a temperature of 50 °C for 48 h. Subsequently, the sample surface was sprayed with gold to enhance its electrical conductivity (30 s for each spray, 5 sprays in total). Each sample was magnified to 1000× and 5000× for microscopic quantitative analysis and microscopic qualitative analysis, respectively. Photoshop (PS) was used to conduct image pre-processing, as an original image may contain defects such as uneven background and low contrast. In order to achieve better segmentation effects and to more accurately calculate the distribution of particles and pores, pre-processing is thus necessary to improve the imaging quality. The main steps of this are: original image → remove tab bar → remove uneven background → spatial contrast enhancement → frequency domain contrast enhancement. In this paper, an image brightness uniformization method was used to reduce the background grey level, and the image enhancement method uses grey level transformation and filtering. MATLAB was then used to conduct image segmentation, and a gradient-based watershed algorithm was used to obtain binary map. On this, white represents particles while black represents pores. Image Pro Plus (IPP) was then used to measure particle size and pore size in binary maps; however, as IPP software directly measures the black sections of such binary maps by default, the target binary map was therefore inverted.

A Fourier transform infrared spectrometer (Nicolet iS50, Thermo Fisher Scientific (China) Co., Ltd., Waltham, Massachusetts USA, with resolution: 2.25px-1; wavenumber accuracy: 0.25px-1; linearity: less than 0.07% (ASTM1421); and spectral range: host 7800-8750px-1, diamond ATR 5000-2000px-1) was used to conduct FITR testing; attenuation total reflection Fourier infrared spectrum analysis (ATR-FTIR) was adopted as the analytical method.

## 3. Results and Discussion

### 3.1. Deterioration Characteristics of the Engineering Properties of Pb-CSCSs under Long-Term Repeated Freeze-Thaw Cycles

#### 3.1.1. UCS (q_u_)

**Figure 3:**
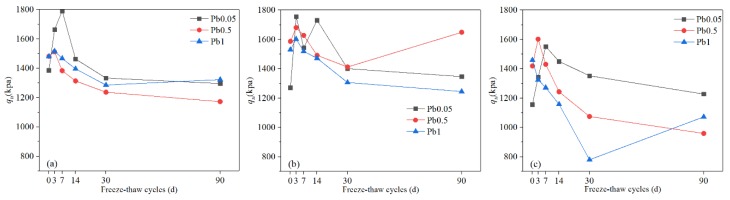
The UCS of Pb-CSCSs with the same compound binder and different contaminant contents: (**a**) C2.5S5F5; (**b**) C5S2.5F2.5, and (**c**) C5S5.

From Figure 3, it can be seen that the UCS of all samples with high contaminant content (0.5% or 1%) before freezing and thawing were higher than those of samples with low contaminant content (0.05%). This indicates that Pb^2+^ contributes to the strength of the solidified soils to a certain degree due to the promotion of the hardening reaction caused by Ca^2+^, obtained from the displacement reaction between lead ions and calcium ions in the gels [64,65].

**Figure 4:**
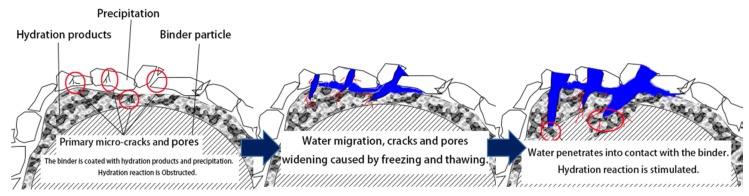
Schematic diagram: Activation of hydration by freeze-thaw cycles.

The curves also show that the UCS of Pb-CSCSs were improved in different degrees in the early stage of the freeze-thaw process (0 to 3d or 0 to 7d), due to extensive Pb^2+^ reactions with the hydroxide generated by binders forming insoluble precipitation, which covers the surface of the binders and makes these unable to make full contact with water, hindering the formation of the hydration products [29,66] which are the main sources of UCS. Thus, for freeze-thaw cycles of 0 to 3d or 0 to 7d, the water in the soil condenses into ice lenses under the action of freezing, causing volume expansion and great pressure within the pores [31], causing the sediments and particles cemented together by hydration to break apart and become dispersed again [33]. The migration and physical state changes of water caused by freezing and thawing [23,30,67] rupture the coating of the precipitate and hydration products so that the water can infiltrate into the gap and produce hydration products after reacting with binders (Figure 4). Although the freezing-thawing cycle damages the soil structure to a certain degree, it was not the main factor dominating the UCS where the number of freeze-thaw cycles was relatively small.

The increased rates of UCS of C2.5S5F5 solidified soils were 29.3% (Pb^2+^ 0.05%, 0 to 7d), 2.1% (Pb^2+^ 0.5%, 0 to 3d), and 2.5% (Pb^2+^ 1%, 0 to 3d), respectively; for C5S2.5F2.5 solidified soils, the measures were 38.1% (Pb^2+^ 0.05%, 0 to 3d), 5.9% (Pb^2+^ 0.5%, 0 to 3d), 4.7% (Pb^2+^ 1%, 0 to 3d), respectively; while for C5S5 solidified soils, these were 34.2% (Pb^2+^ 0.05%, 0 to 7d), 12.9% (Pb^2+^ 0.5%, 0 to 3d), −9.2% (Pb^2+^ 1%, 0 to 3d), respectively. As Pb^2+^ content increased to 0.5%, the increase rate of UCS in the early stage sharply decreased compared where Pb^2+^ content was 0.05%, though it increased little (Figure 3a,b) and showed a tendency to even decrease (Figure 3c) with freeze-thaw cycles for 1% Pb^2+^ content. This indicates that, in addition to creating a sediment that physically blocks hydration, Pb^2+^ also has the retardation effects on the ion exchanges and agglomerations and pozzolanic reactions in binders that also caused a loss in UCS [28,54]. Therefore, the higher the pollutant content, the more precipitates formed, as well as the more excess Pb^2+^ dissolved in pore water and the more serious resulting retardation effect on ion exchanges and agglomerations and pozzolanic reactions, all of which causes a reduction of the gains from UCS. As the freeze-thaw cycles continue, the control factor of UCS transforms from the hydration excitation effect to the structural damage to Pb-CSCSs caused by freezing and thawing, which results in a continuous decrease in UCS throughout the entire freeze-thaw process of Pb-CSCSs with higher Pb^2+^ content and in the later stages of samples with lower Pb^2+^ content. The fluctuations of the UCS of C5S2.5F2.5Pb0.5 and C5S2.5F2.5Pb0.05 (Figure 3b) further confirmed that the hydration hindrance effect caused by the precipitates, the stimulation of hydration, and the structural deterioration effect caused by freeze-thaw cycles alternately play leading roles in soil strength, and that freeze-thaw cycles will eventually have a deterioration effect on the strength of Pb-CSCSs.

**Figure 5:**
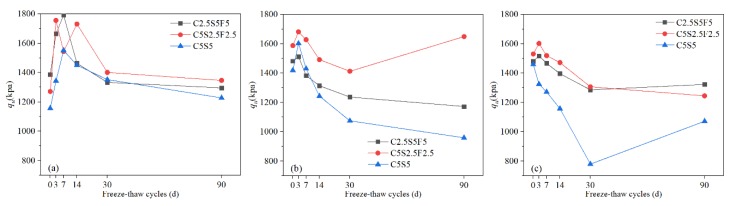
The UCS of Pb-CSCSs with the same contaminant content and different compound binders: (**a**) 0.05% Pb^2+^; (**b**) 0.5% Pb^2+^, and (**c**) 1% Pb^2+^.

After 90 days of the freeze-thaw process, the UCS of Pb-contaminated soils solidified with C5S5 were dramatically reduced, by 32.4% (0.5% Pb^2+^) and 26.5% (1% Pb^2+^). Figure 5 shows that the UCS of Pb-contaminated soils solidified with C5S5 was generally smaller than those of C2.5S5F5 or C5S2.5F2.5 due to the increase in lime content leading to more precipitate being generated, which hinders hydration. Additionally, after the fly ash was added, Ca(OH)_2_ reacts with the active oxide in the glass phase of the fly ash particles and forms gels such as calcium aluminate hydrate (CAH) and calcium silicate hydrate (CSH), which encapsulate the particles and fill the pores, resulting in significant increases in soil strength [68,69]. This proves that the addition of fly ash could effectively resist the continuous deterioration of freeze-thaw actions. In actual engineering use, the addition of a certain amount of fly ash is therefore beneficial to improving the strength and stability under long-term freeze-thaw cycles.

Additionally, it was found that the UCS of all Pb-CSCSs throughout the freeze-thaw cycle reached up to 800 to 1800 kpa, much higher than the British design UCS value of 0.35 Mpa [33,70], indicating that the strength of the contaminated soil after compound curing was significantly improved. The small gains and decreases in UCS of C2.5S5F5Pb0.05 (−6.5%), C5S2.5F2.5Pb0.05 (5.9%) and C5S5Pb0.05 (6.1%) suggested that Pb-CSCSs had great resistance to freeze-thaw damage when Pb^2+^ content was relatively low. This could again be attributed to the generation of contaminated precipitates, which hinders the normal hydration of the binders so that a large amount of the binder added to the soil does not fully participate in hydration. Although freeze-thaw cycles damage the structure of the soil and reduced its strength, they also trigger the migration of water, stimulating the hydration of the binders, hence, the soil structure is strengthened again, demonstrating additional resistance to freeze-thaw degradation over a period of time.

Nonparametric hypothesis testing (Supplementary Table S1) showed that the significance of the UCS of Pb-contaminated soils solidified with C2.5S5F5 and C5S5 was greater than 0.05 (0.115, 0.223, respectively). This means that, under long-term freeze-thaw cycles, the treatment effect is not significantly affected by Pb^2+^ content changing from 0.05% to 1%. From the average rank of C5S2.5F2.5 solidified Pb-contaminated soils with three Pb^2+^ contents shown in the panel histogram (Supplementary Figure S1), it can be seen that long-term freeze-thaw cycles have the least influence on the UCS of C5S2.5F2.5Pb0.5, and the most on that of C5S2.5F2.5Pb1, with C5S2.5F2.5Pb0.05 somewhere in between. Furthermore, pairwise comparisons (Supplementary Table S2) reveal that the adjusted significance of the Pb^2+^ contents, 0.05% to 0.5% and 0.5% to 1%, were larger than 0.05 (1.000, and 0.250, respectively), indicating that, using UCS as the indicator, there were no significant differences in the curing effect of C5S2.5F2.5 when the Pb^2+^ content changed from 0.05% to 0.5% or 0.5% to 1%, suggesting that there could be a critical Pb^2+^ content that causes a significant change in its repair efficacy.

#### 3.1.2. Deformation modulus (E_50_)

**Figure 6:**
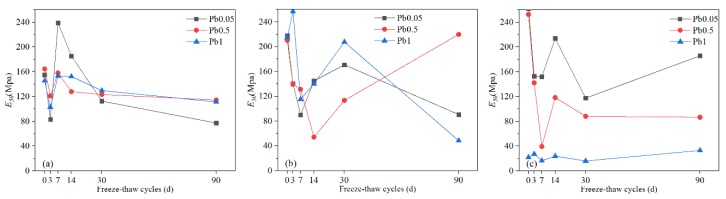
The *E_50_* values of Pb-CSCSs with the same compound binder and different contaminant contents: (**a**) C2.5S5F5; (**b**) C5S2.5F2.5, and (**c**) C5S5.

**Figure 7:**
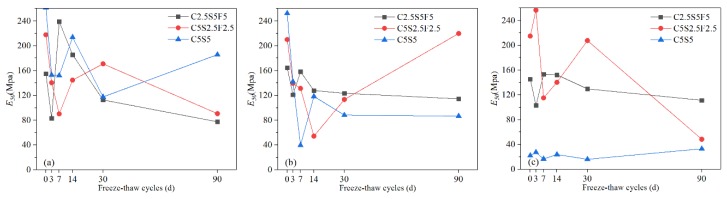
The *E_50_* of Pb-CSCSs of the same contaminant content and different compound binders: (**a**) 0.05% Pb^2+^; (**b**) 0.5% Pb^2+^, and (**c**) 1% Pb^2+^.

Figure 6 shows that the hindrance effect on hydration and the various reactions mentioned above caused by Pb^2+^, the stimulation of hydration, and the structural deterioration effect on Pb-CSCSs caused by freeze-thaw cycles all lead to *E_50_* exhibiting large fluctuations; overall, in these experiments, it tended to become smaller. The *E_50_* of the three different Pb^2+^ contaminated soils solidified with C2.5S5F5 (Figure 6a) were relatively close to each other; the *E_50_* values of C2.5S5F5Pb0.5 and C2.5S5F5Pb1 were particularly stable, showing good resistance against long-term freeze-thaw cycles. This may be attributed to the higher lime and Pb^2+^ content leading to the formation of more Pb(OH)_2_. Cement and fly ash are more tightly wrapped by precipitation products, resulting in incomplete hydration, and the freeze-thaw cycle thus unlikely stimulates further hydration of cement and fly ash. Thus, these *E_50_* values were relatively stable and close. The curves in Figure 6b fluctuate greatly and show no typical characteristics, while the *E_50_* of C5S2.5F2.5Pb0.5 shows an increasing trend in the later period in contrast to the trends of the other two samples. The reduction of lime content resulted in less Pb(OH)_2_ being produced, and the freeze-thaw cycle effectively stimulated further hydration of cement and fly ash. The two effects of freeze-thaw cycles, damage to soil structure, and promotion to hydration, randomly dominated during freeze-thaw processes, thus causing *E_50_* to fluctuate greatly. It could also be this randomness that caused the increase in *E_50_* of C5S2.5F2.5Pb0.5. Figure 6c and Figure 7 show that the *E_50_* of the C5S5 solidified Pb-contaminated soil deteriorated dramatically with the increase of Pb^2+^ content; in particular, the *E_50_* of C5S5Pb1 was much smaller than those of C5S5Pb0.05 and C5S5Pb0.5 throughout the process. Furthermore, the *E_50_* values of Pb-CSCSs were much more sensitive changes in Pb^2+^ content, indicating that the lack of fly ash resulted in weaker soil structure. According to Nalbantoglu et al. [71,72], fly ash provides stable exchangeable cations, such as Ca^2+^,Al^3+^, and Fe^3+^, which promote flocculation of the clay particles, thus reducing the plasticity index, activity, and swell potential of soil. Furthermore, time-dependent cementation processes (pozzolanic reactions) result in formation of cementing compounds with high strength and low volume variation. In the authors’ previous study [57], 2.5% was found to be the favourable content level in compound binders for *E_50_*. More sediments were generated due to the significant difference in CaO content (84.23% in lime and 5.73% in fly ash), which prevented further hydration of the binders by freezing and thawing. This is consistent with previous analyses.

It is clear from Supplementary Table S3 that the significance of the *E_50_* values of C2.5S5F5 and C5S2.5F2.5 solidified Pb-contaminated soils were greater than 0.05 (0.607, and 0.223, respectively), showing that, under long-term freeze-thaw cycles, the treatment effect is not significantly affected by the Pb^2+^ content changing from 0.05% to 1%. Figure S2 shows that long-term freeze-thaw cycles have the least influence on the *E_50_* of C5S5Pb0.05 and the most on that of C5S5Pb1, with that of C5S5Pb0.5 somewhere in between. Furthermore, pairwise comparisons (Supplementary Table S4) show the adjusted significances of the Pb^2+^ content moving from 0.05% to 0.5% and 0.5% to 1% were greater than 0.05 (both 0.250), indicating that, taking *E_50_* as the indicator, there were no significant differences in the curing effect of C5S5 when the Pb^2+^ content changed from 0.05% to 0.5% or 0.5% to 1%, suggesting that there could be a critical Pb^2+^ content that causes a significant change in solidification/stabilization efficacy.

#### 3.1.3. Internal Friction Angle (φ)

**Figure 8:**
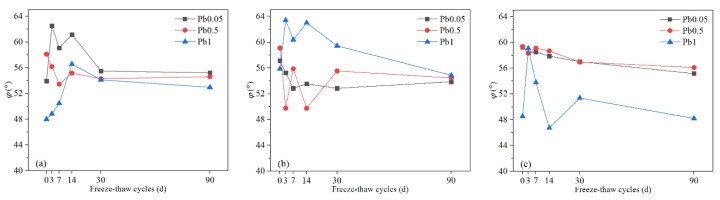
The *φ* values of Pb-CSCSs with the same compound binder and different contaminant contents: (**a**) C2.5S5F5; (**b**) C5S2.5F2.5, and (**c**) C5S5.

The soil after solidification resembled coarse-grained soil, whose internal friction angle is affected by many factors, including density, particle size gradation, particle shape, and mineral composition. From a macroscopic perspective, with 1% Pb^2+^, the *φ* of Pb-contaminated soils solidified with C2.5S5F5 (Figure 8a) and C5S5 (Figure 8c) were generally smaller than the *φ* when the Pb^2+^ content was 0.05% or 0.5%. This reflects that increasing the content of Pb^2+^ leads to stronger hindrance to the hydration of binders, affecting the engineering properties of Pb-CSCSs under long-term freeze-thaw cycles. Additionally, before freezing and thawing, an improvement in the *φ* when Pb^2+^ content was 0.5% over Pb^2+^ contents of 0.05% or 1% indicated there could be a critical content of Pb^2+^ (between 0.5% and 1%) that results in the enhancement function of Pb^2+^ on *φ* being converted to a degradation function.

In all the tested samples, after 90 days of freezing and thawing, the maximum decrease in *φ* was 7.9% (C5S2.5F2.5Pb0.5) and the maximum increase was 10.4% (C2.5S5F5Pb1). The *φ* of C5S2.5F2.5Pb1 was generally greater than that of C5S2.5F2.5Pb0.05 or C5S2.5F2.5Pb0.5 (Figure 8b). Precipitation was broken by the freeze-thaw cycles when the Pb^2+^ content was high, which improved the gradation and shape of particles, enhancing the friction of the shear surface. In contrast, when the Pb^2+^ content was low, its hindrance effect on the hydration reaction was weakened such that the binders already had a higher degree of hydration before freezing and thawing, leading to damage caused by freezing and thawing dominating the soil structure.

**Figure 9:**
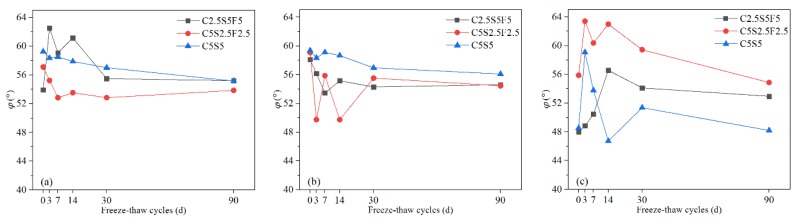
The *φ* values of Pb-CSCSs with the same contaminant content and different compound binders: (**a**) 0.05% Pb^2+^; (**b**) 0.5% Pb^2+^, and (**c**) 1% Pb^2+^.

There were small changes in the *φ* of Pb-CSCSs after a certain number of freeze-thaw cycles (30d). In particular, the *φ* values of C5S5Pb0.05 and C5S5Pb0.5 were relatively stable during the entire long-term freeze-thaw process (Figure 8c and Figure 9a,b), showing good resistance to long-term freeze-thaw cycles. This indicates that when the content of Pb^2+^ is low (0.05%, 0.5%), and the content of lime and fly ash are 5% and 0%, respectively, the binders are highly hydrated before freezing and thawing so that *φ* is stable and slightly reduced under the comprehensive influence of the continuous deterioration and improved particle grading and shape caused by freeze-thaw cycles. In addition, in all three kinds of Pb-CSCSs, the variation characteristics in *φ* were similar when the pollution levels were 0.05% and 0.5%, indicating that, under the combined effect of the pollutants in this content range and the freeze-thaw cycle, the geometric arrangement and shape of soil particles did not change significantly, in agreement with Li et al. [73].

However, when the Pb^2+^ content increased to 1%, the friction angle was significantly smaller than in C5S5Pb0.05 and C5S5Pb0.5. This might be caused by the generation of a large amount of Pb(OH)_2_, which would prevent further cement hydration. Although the friction angle increased significantly when the freeze-thaw cycle increased from 0d to 3d, this may be due to more precipitation and the crushing of solidified particles by freezing and thawing leading to an increase in the internal friction angle. With the increase in the number of freeze-thaw cycles, more precipitation would thus have prevented hydration from being stimulated by the freeze-thaw cycle, and thus the continuous degradation of the freeze-thaw cycle would predominate, resulting in a significant decrease in the internal friction angle and making it significantly smaller than those of the other two Pb-CSCSs. The occurrence of large differences in internal friction angles once again suggests that a critical content level may exist between 0.5% and 1%.

The nonparametric hypothesis tests (Supplementary Table S5) showed that the significances of the *φ* values of C5S2.5F2.5 and C5S5 solidified Pb-contaminated soils were greater than 0.05 (both 0.115), suggesting that, under long-term freeze-thaw cycles, the treating effect was not significantly affected by Pb^2+^ content changing from 0.05% to 1%. Supplementary Figure S3 shows that the long-term freeze-thaw process also has the least influence on the *φ* of C2.5S5F5Pb0.05 and the most on that of C2.5S5F5Pb1, with C2.5S5F5Pb0.5 somewhere in between. Pairwise comparisons (Table S6) showed the adjusted significances of the Pb^2+^ content from 0.05% to 0.5% and 0.5% to 1% were greater than 0.05 (both 0.447), suggesting that, taking *φ* as the indicator, there were no significant differences in the curing effect of C2.55S5F5 when Pb^2+^ content changed from 0.05% to 0.5% or 0.5% to 1%. Again, this may suggest that there could be a critical Pb^2+^ content that causes a significant change in solidification/stabilization efficacy.

#### 3.1.4. Cohesion (c)

**Figure 10:**
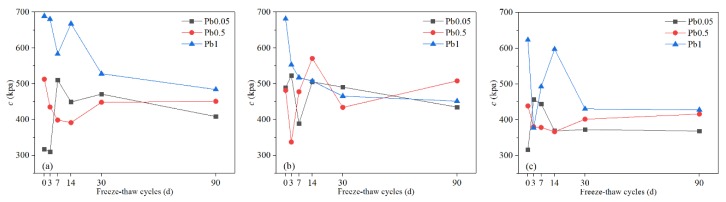
The ***c*** of Pb-CSCSs with the same compound binder but different contaminant contents: (**a**) C2.5S5F5; (**b**) C5S2.5F2.5, and (**c**) C5S5.

**Figure 11:**
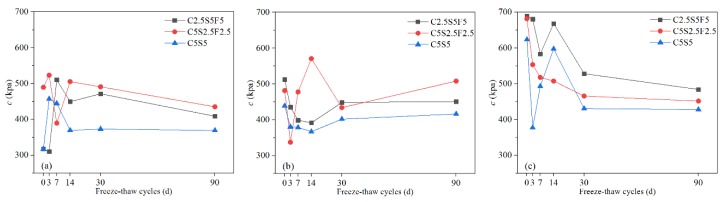
The ***c*** of Pb-CSCSs with the same contaminant content but different compound binders: (**a**) 0.05% Pb^2+^; (**b**) 0.5% Pb^2+^, and (**c**) 1% Pb^2+^.

Cohesion depends on the distance between particles, the number of contact points per unit surface area of the particles, and various physical and chemical forces between the particles, including the Coulomb force (electrostatic force), van der Waals force, and cementation force. Fluctuations of the cohesion of Pb-CSCSs were observed as seen in Figure 10, and further observations showed that the cohesion generally improved with the increase of Pb^2+^ content, especially when the Pb^2+^ content of Pb-CSCSs was 1% (Figure 11c), where the cohesion was almost always greater than that of Pb-CSCSs with lower Pb^2+^ content (0.05% and 0.5%) throughout the freeze-thaw process. This can be attributed to the adsorption that is the main mechanism of Pb^2+^ solidification [74,75], as soil particles become negatively charged due to edge breakage, ion exchange, and dissociation of hydrogen in the hydroxyl group. A diffuse electrical double layer (DDL) thus exists between the soil particles, and its thickness is an important factor affecting the cohesion of the soil. The appearance of Pb^2+^ in pore water fluid reduced the thickness of hydrated film and DDL between the soil particles, resulting in reduced osmotic repulsion and increased van der Waals force attraction among the clay [67,73,76]. Hence, the particles were bonded more firmly, contributing to increased cohesion. Additionally, the formation of pollutant precipitates filled the pores between the particles to a certain extent, reducing the distance between the particles. The contact points between the particles increased, macroscopically generating a significant improvement in cohesion [77]. The cohesion decreased sharply in all three kinds of solidified contaminated soil with increases in the freeze-thaw cycle when Pb^2+^ content was 1% (Figure 11c), however, and it was significantly lower than the initial value after 90 days of freeze-thaw. In contrast, when the Pb^2+^ levels were 0.05% (Figure 11a) and 0.5% (Figure 11b), the cohesion of the samples was relatively close during the freeze-thaw cycles, and the final cohesion was only slightly decreased, or even increased, from the initial value. Although increased Pb^2+^ can greatly improve initial cohesion, a large amount of precipitation seriously impedes the activation of hydration in later freezing-thawing cycles. The degradation of cohesion caused by freezing-thawing cycles thus dominates. Moreover, even with different contamination levels, the cohesion of samples solidified by the same compound binder tended to level off to an equal value. The reason for this trend could be that the shape, size, and spacing of soil particles tends to be more homogeneous as the freeze-thaw process progresses, which causes similar cohesion.

In nonparametric hypothesis tests (Table S7), the significances of the *c* values of C5S2.5F2.5 and C5S5 solidified Pb-contaminated soils were greater than 0.05 (0.311, and 0.136, respectively), suggesting that, under the long-term freeze-thaw cycles, the treating effect is not significantly affected by Pb^2+^ contents changing from 0.05% to 1%. Supplementary Figure S4 shows that long-term freeze-thaw cycles have the least influence on the *c* of C2.5S5F5Pb1, and the most on that of C2.5S5F5Pb0.05, with C2.5S5F5Pb0.5 in between. Furthermore, pairwise comparisons (Table S8) shows the adjusted significance of Pb^2+^ content changing from 0.05% to 0.5%, was greater than 0.05 (1.00), indicating that, taking *c* as the indicator, there were no significant differences in the curing effect of C2.55S5F5 when the Pb^2+^ content changed from 0.05% to 0.5%. Again, there could be a critical Pb^2+^ content between 0.5% and 1% that causes a significant change in solidification/stabilization efficacy.

#### 3.1.5. Permeability Coefficient (k)

**Figure 12:**
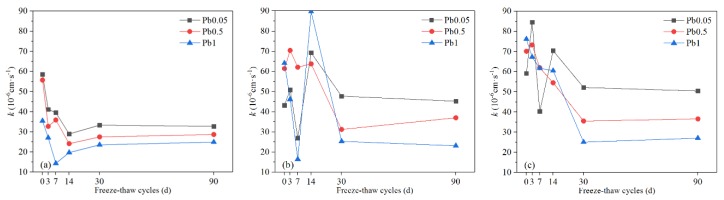
The *k* values of Pb-CSCSs with the same compound binder and different contaminant contents: (**a**) C2.5S5F5; (**b**) C5S2.5F2.5, and (**c**) C5S5.

As shown in Figure 12, the permeability coefficient of Pb-CSCSs fluctuated and finally decreased over increases in freeze-flow cycles. The variation characteristics of the permeability coefficients of Pb-CSCSs with three different Pb^2+^ contents solidified with C2.5S5F5 were very similar throughout (Figure 12a). In the initial stages of freeze-thaw cycles, the permeability coefficient gradually decreased as the freeze-thaw cycles increased, then on reaching a certain point (7d or 14d), it began display a slight upward trend and eventually levelled out. This trend could be attributed to some pores being filled with hydration products due to the stimulation of freeze-thaw cycles of hydration in the binders. Moreover, when the particles were broken by freezing and thawing, the finer particles formed block some percolation channels, thereby macroscopically decreasing the permeability coefficient in the early stages. Subsequently, as the freeze-thaw process continues, the damage done by freeze-thaw cycles dominates the structure of Pb-CSCSs, resulting in larges pores and multiple small seepage channels, which lead to larger but more stable permeability values. The reason the permeability coefficient of C5S2.5F2.5 solidified soils (Figure 12b) fluctuates greatly is similar: the freezing and thawing causes the solidified soil structure to be broken into larger particles, forming open seepage channels, with these larger particles broken up again so that the resulting fine particles block the percolation passages.

**Figure 13:**
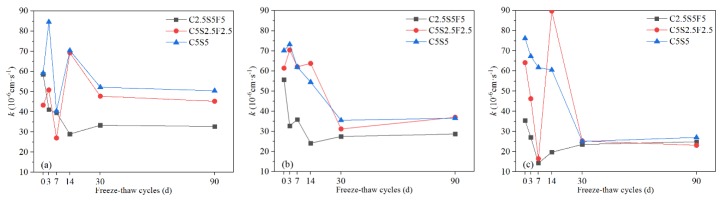
The *k* values of Pb-CSCSs with the same contaminant content and different compound binders: (**a**) 0.05% Pb^2+^; (**b**) 0.5% Pb^2+^, and (**c**) 1% Pb^2+^.

The permeability coefficients of all the Pb-CSCSs with higher Pb^2+^ content were smaller than those with lower Pb^2+^ contents after long-term freeze-thaw processes, in direct contrast to Li et al. [73], who suggested that the permeability of uncured remediation contaminated soil is positively correlated with lead concentration. The difference may lie in the presence of binders in the Pb-CSCSs. Higher levels of Pb^2+^ in soil contribute to more precipitation after soil has been treated, which fills the pores. Further, when the initial content of Pb^2+^ increases to exceed the soils precipitation and adsorption capacity, excessive Pb^2+^ crystallises in the pores [64], leading to a decrease in the permeability of the solidified soil. The permeability coefficient of Pb-CSCSs was effectively reduced by increasing the content of fly ash in the compound binders (Figure 13). Excessive hydration products produced by larger amounts of binders lead to strong cementation and compaction of soil particles, widening the leakage channels and finally increasing permeability. However, compared with cement and lime, fly ash has a low degree of hydration and produces the fewest gelatinous hydration products were produced, thus reducing its effect on the permeability of Pb-CSCSs [57].

Supplementary Table S9 shows that the significance of the *k* value of the C5S2.5F2.5 solidified Pb-contaminated soils was greater than 0.05 (0.607), suggesting that under long-term freeze-thaw cycles, the treating effect was not significantly affected by Pb^2+^ content changing from 0.05% to 1%. Figure S5 shows that the long-term freeze-thaw process has the least influence on the *k* values of C2.5S5F5Pb0.05 and C5S5Pb0.05, and the most on that of C2.5S5F5Pb1 and C5S5Pb1, with C2.5S5F5Pb0.5 and C5S5Pb0.5 somewhere in between. Pairwise comparisons (Supplementary Table S10) showed the adjusted significances of Pb^2+^ content changing from 0.05% to 0.5% and 0.5% to 1%, were greater than 0.05, indicating that, taking *k* as the indicator, there were no significant differences in the curing effect of C2.55S5F5 and C5S5 when the Pb^2+^ contents changed from 0.05% to 0.5% or 0.5% to 1%. Again, there may thus be a critical Pb^2+^ content that causes a significant change in solidification/stabilization efficacy.

### 3.2. Micro-Mechanism

#### 3.2.1. Development of Soil Porosity (CT)

**Figure 14:**
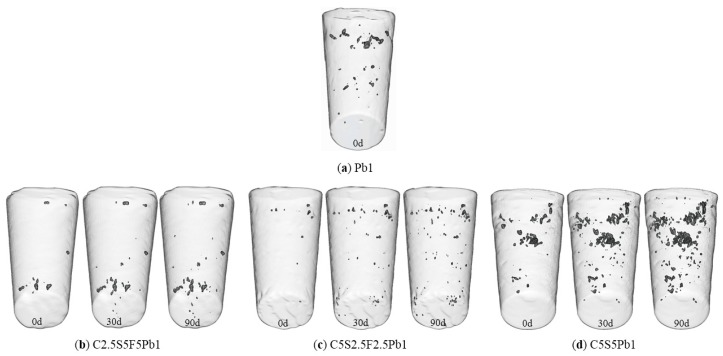
Three-dimensional perspectives of specimens before and after freeze-thaw process.

Unsolidified Pb-contaminated soil (Pb1) has many large pores and a certain quantity of “large volume” pores (Figure 14a), indicating that the lead-contaminated soil has large structural defects without solidification. The pores of C2.5S5F5Pb1 and C5S2.5F2.5Pb1 were significantly reduced (Figure 14b,c), suggesting that structural defects of the specimens were significantly improved, a process conducive to improving soil strength and resistance against deformation.

The number of pores in the three Pb-CSCSs increased with increasing freeze-thaw cycles, but this increase was balanced by the amount of “small” pores, explaining the micro-mechanisms by which the UCS and the deformation modulus of Pb-CSCSs are gradually degraded and showing good resistance to freeze-thaw cycles. However, the pores of C5S5Pb1 solidified lead contaminated soil were much more prominent than that those of Pb1, C2.5F5S5Pb1, and C5S2.5F2.5Pb1 under the same freezing and thawing cycles (Figure 14d). This suggests that the solidified contaminated soil tends to produce additional large aggregates and to form larger pores when the content of lime is relatively high due to the specific solidification mechanisms of lime [78]. This intuitively reflects the micro-mechanisms results of *qu*, *E_50_* and the *c* of C5S5Pb1 being relatively smaller and the *k* of C5S5Pb1 being larger than those of the other two kinds of Pb-CSCSs (Figure 5c, Figure 7c, Figure 11c, and Figure 13c) under the same freeze-thaw cycles. The addition of a small amount of lime (2.5%) and an appropriate amount of fly ash (2.5% or 5%) can thus help avoid the production of structural defects; effectively improving the engineering properties and resistance of Pb-CSCSs against long-term repeated freeze-thaw processes.

#### 3.2.2. Correlations between UCS and Particle Size, K and Pore Size (SEM)

**Figure 15:**
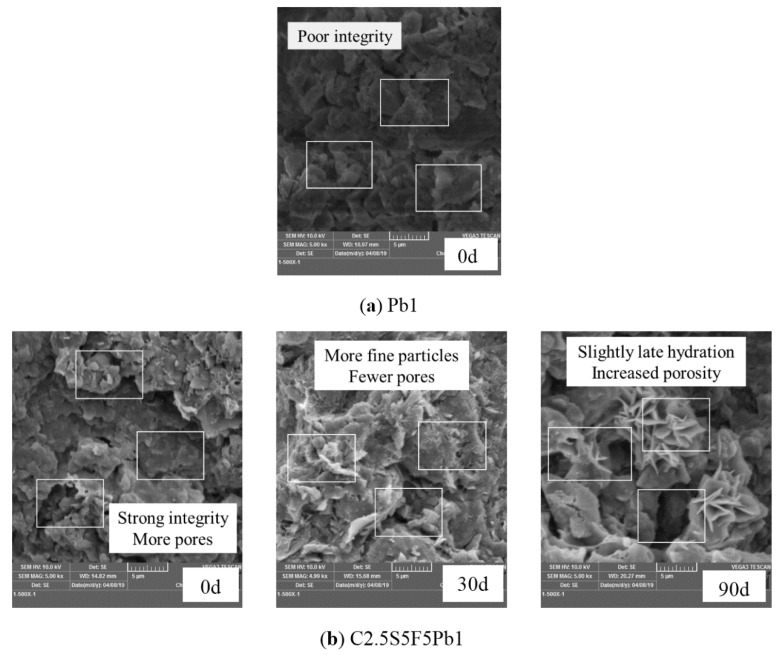

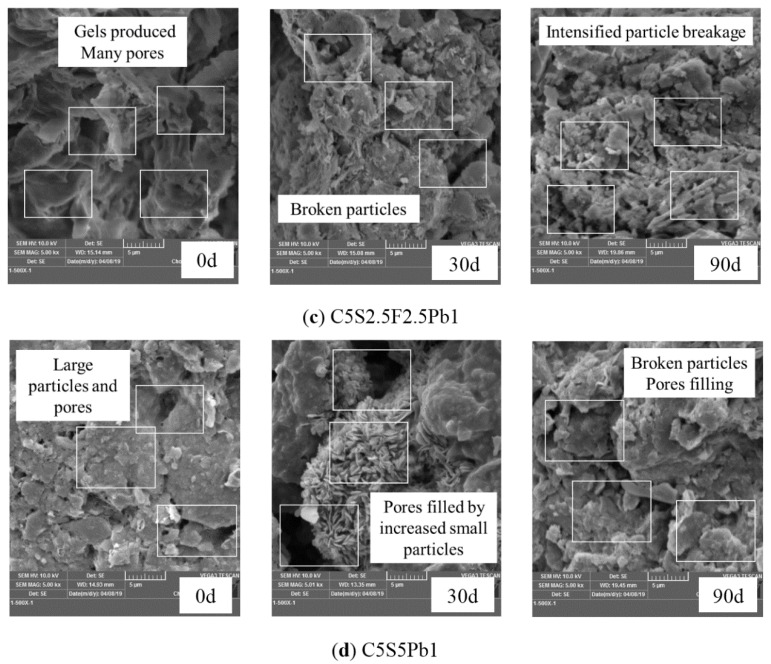
SEM images of Pb-contaminated soil and Pb-CSCSs under freeze-thaw cycles.

By comparing the SEM images of the Pb-contaminated soil (Figure 15a) with those of the three kinds of Pb-CSCSs prior to freezing and thawing (Figure 15b–d), it is clear that the integrity of soil is significantly improved where soil particles are closely combined by hydration products; thus, the strength of all three kinds of Pb-CSCSs were effectively improved. The formation of large particles also created additional larger connected pores in the solidified contaminated soil, resulting in enhanced soil permeability, increasing the leaching risk of pollutants. All Pb-CSCSs showed a gradual breaking up of consolidated particles, displayed on the macro level as a gradual decrease in their UCS with increased freeze-thaw cycles.

New hydration products (C-S-H gel) [79] were observed, as seen in Figure 15b, under a 90d freeze-thaw cycle, which proved that pollutant precipitates block the hydration reaction while the freeze-thaw cycles promote it. This directly reflects the micro-mechanism of improvement of the UCS of C2.5S5F5Pb1 on freezing and thawing for 90d compared to freezing and thawing for 30d (Figure 5c). In addition, on a 30d freeze-thaw cycle, tiny broken particles, produced by freeze-thaw cycles and hydration, filled the pores, making the soil structure denser. However, when the freeze-thaw cycles increased to 90d, damage to the soil structure was clear, and obvious large pores were generated. These results are in agreement with the various characteristics of the permeability coefficient of C2.5S5F5Pb1 as represented in Figure 13c.

Figure 15c shows that the number of tiny broken particles in the soil obviously increased with the increase in freeze-thaw cycles, and that their size of them tended to become homogenized; many tiny pores were formed due large pores being filled by those tiny particles. This shows more clearly that freeze-thaw cycles have a crushing effect on the consolidated particles in Pb-CSCSs, reflecting the macro aspect of permeability coefficient gradually decreasing and tending to become stable.

**Figure 16:**
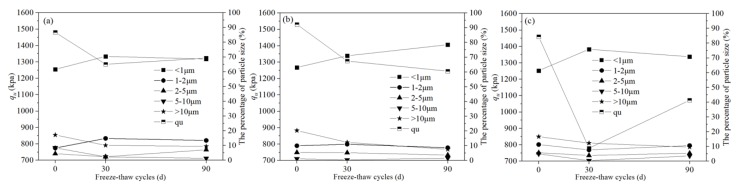
The *q_u_* and particle size distribution of Pb-CSCSs: (**a**) C2.5S5F5; (**b**) C5S2.5F2.5, and (**c**) C5S5.

Based on the relationship between UCS and particle size distribution in Pb-CSCSs (Figure 16), it can be seen that in the early stage of freeze-thaw process (0 to 30 d), the number of particles larger than 2 μm in C2.5S5F5Pb1 and C5S2.5F2.5Pb1 decreased smaller than 2 μm increased (Figure 16a,b). In C5S5Pb1, all particles larger than 1 μm decreased, while those smaller than 1 μm increased (Figure 16c), which indicates a more intense crushing effect. At the same time, the UCS of all three kinds of Pb-CSCSs dropped significantly under 30d freeze-thaw cycles, with the UCS of C5S5Pb1 decreasing most sharply. This indicates that the larger particles in Pb-CSCSs were broken under freeze-thaw cycles, reducing the UCS; the smaller the maximum particle size of the broken particles, the more dramatically the UCS deteriorated.

When freeze-thaw cycles were 30 to 90 d, particles with particle sizes of 1–5 μm in C5S2.5F2.5Pb1 continued to decrease and the number of particles with particle size 5–10 μm showed a certain degree of rebound (Figure 16b) due to the tiny particles were partly cemented together, as well as the breakage of larger particles. However, since the freezing and thawing played a dominant role in the crushing of soil particles, the content of particles larger than 10 μm continued to decrease, while the content of particles smaller than 1 μm slowly increased, causing the UCS of C5S2.5F2.5Pb1 to continue to drop to a small extent.

For C2.5S5F5Pb1 and C5S5Pb1, the intensified hydration of binders leads to a reduction in particles smaller than 1 μm, and the proportions of particles of 2–5 μm (Figure 16a) and 1–10 μm (Figure 16c) showed substantial increases. This caused the UCS of the two to improve in the later stages of freeze-thaw cycles, with the UCS of C5S5Pb1 more significant.

**Figure 17:**
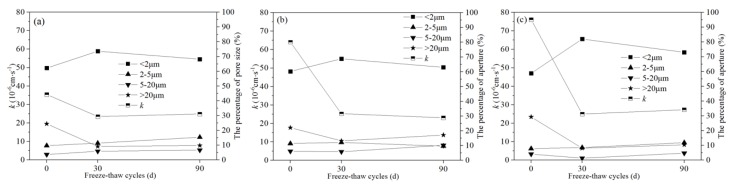
The *k* and pore size distribution of Pb-CSCSs: (**a**) C2.5S5F5; (**b**) C5S2.5F2.5, and (**c**) C5S5.

As shown in Figure 17, after the completion of curing of the three Pb-CSCSs, the proportion of pores larger than 20 μm was relatively increased, especially in C5S5Pb1, resulting in greater permeability. For 30 d freeze-thaw cycles, the larger pores in the soil were filled, forming more small pores due to the combined effects of the hydration of binders and large particle breakage caused by freeze-thaw cycles, causing hydraulic conductivity to be drastically reduced, especially in C5S5Pb1, consistent with the results presented in Figure 12. As the freeze-thaw process continued (30–90 d), the activated hydration and particle breakage in C2.5S5F5Pb1 and C5S5Pb1, resulted in a reduction in the number of tiny pores smaller than 2 μm and a small increase in the proportion of the larger pores larger than 2 μm, which eventually led to a slight increase in hydraulic conductivities (Figure 17a,c). However, the particles in C5S2.5F2.5Pb1 continued to be broken, which increased the number of pores larger than 20 μm (13.23% to 17.13%), while the number of pores smaller than 5 μm decreased significantly (80.93% to 72.66%), causing its permeability coefficient to show a tendency towards a slow decrease (Figure 17b).

#### 3.2.3. Variations of Main Functional Groups (FTIR)

**Figure 18:**
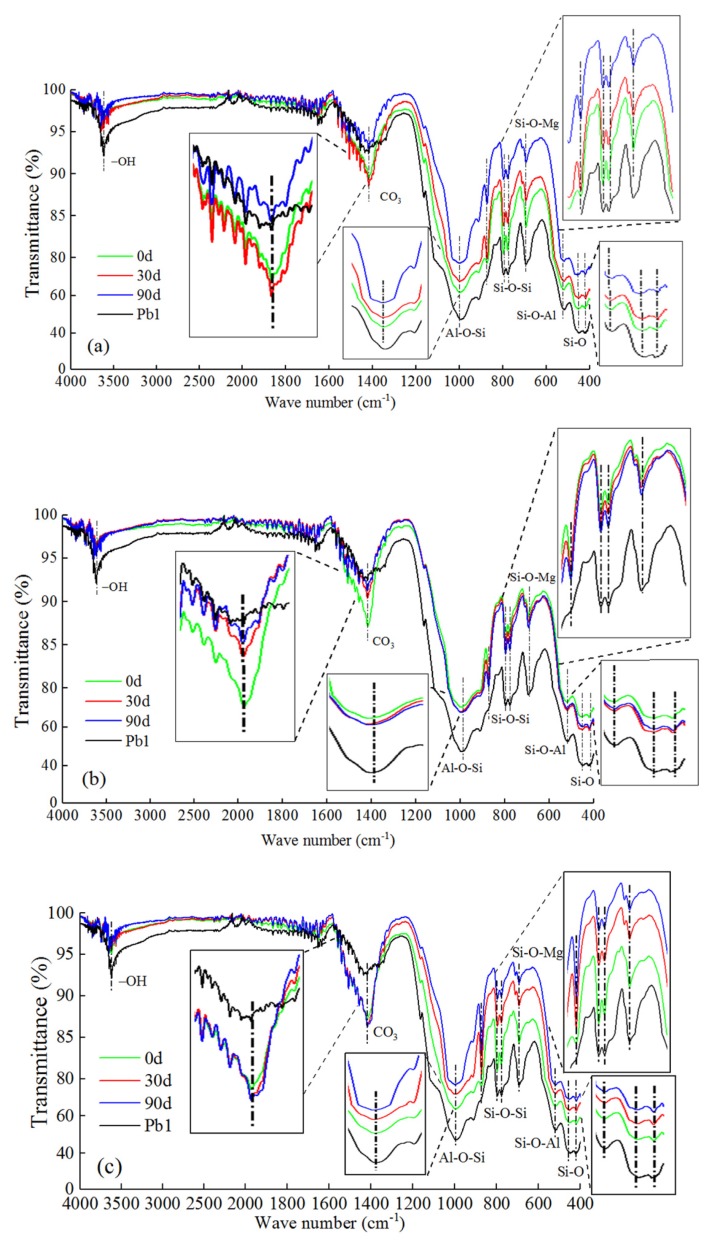
FTIR spectra of Pb-contaminated soil and Pb-CSCSs under freeze-thaw cycles: (**a**) C2.5S5F5Pb1; (**b**) C5S2.5F2.5Pb1, and (**c**) C5S5Pb1.

The FTIR spectra [80,81,82,83,84] of all Pb-CSCSs are similar to that of Pb1 (Figure 18), indicating that the main functional groups of Pb-CSCSs did not change on adding binders. The characteristic peak transmittance caused by the expansion vibration of –OH in the vicinity of 3620 cm^−1^ in the three types of Pb-CSCSs were higher than that of Pb1, which reflects the phenomenon that the adsorbed water and crystal water in the soil transform into structural water on reaction with binders to form CSH. This decrease of water in the environment is conducive to improving the alkaline environment, promoting the precipitation of pollutants and reducing their mobility. In addition, the lead pollutants are wrapped with or adsorbed onto the hydrated gels generated, which also limits the transfer of pollutants, enhancing the soil strength, and achieving solidification and stabilization. The anti-symmetric stretching vibration peak near 1418 cm^−1^ was significantly enhanced when binders were added to Pb-contaminated soil, suggesting that the hydration of cement and lime generates a large amount of calcium hydroxide, which inevitably absorbed carbon dioxide from the air and generated carbonates during the test [85]. Overall, the characteristic peak transmittance of the main functional groups of all three types of Pb-CSCSs increased with increases in the number of freeze-thaw cycles, showing that freeze-thaw cycles have destructive effects on those functional groups.

## 4. Conclusions

In this paper, cement, lime, and fly ash were mixed in various proportions to make compound binders (C2.5S5F5, C5S2.5F2.5, and C5S5) used to compound solidified/stabilized Pb-contaminated soils (Pb-CSCSs). A series of laboratory experiments was then conducted to investigate the variation in the engineering properties of Pb-CSCSs with an increase in freeze-thaw cycles under various Pb^2+^ pollution levels. The main conclusions were as follows:

- UCS (*qu*) was enhanced for short freeze-thaw cycles (3 or 7 d), and the increase of Pb^2+^ resulted in a decrease in its growth. UCS was eventually weakened under long-term freeze-thaw cycles.
- The deformation modulus (*E_50_*) of the Pb-CSCSs with high content of lime and without fly ash was more sensitive to pollutant content, and a high content of Pb^2+^ seriously decreased it.
- Internal friction angle (*φ*) rarely changed after the long-term freeze-thaw process (90d). There could be a critical content of Pb^2+^ (between 0.5% and 1%) that reverses the effect of Pb^2+^ on *φ*.
- Pb^2+^ is beneficial to cohesion (*c*), though the *c* values of those of the soils repaired by the same compound binder at different pollution levels tended to equalise after long-term freeze-thaw.
- The permeability coefficient (*k*) increased after solidifying/stabilizing, and Pb^2+^ and fly ash were beneficial to reducing it. It decreased and tended to stabilise under freezing and thawing.

CT testing showed that the integrity of Pb-contaminated soil was obviously improved after compound repairing. Reducing lime (2.5%) and increasing fly ash (2.5% or 5%) could reduce soil structural defects. SEM testing showed that the number of tiny particles (smaller than 1 μm) and pores (smaller than 2 μm) were distinctly increased when the freeze-thaw cycles reached to 30d, leading to a decrease in UCS and *k*. FTIR testing showed that, although the main functional groups of soil did not change after solidifying/stabilizing, they were destroyed by freezing and thawing.

The engineering properties of most tested samples demonstrated only small gaps between 30 d and 90 d freeze-thaw cycles. Therefore, the values of the engineering properties of various Pb-CSCSs after 30 d of freeze-thaw cycles could be considered for use as a reference to determine the design values of actual engineering projects.

## Figures and Tables

**Table 1:** Basic physical and chemical properties of the soil used in this study.

Properties	Value
Liquid limit W_L_: %	28.6
Plastic limit W_P_: %	16.7
Plastic index I_P_:	11.9
Optimum water content: %	13.65
Maximum dry density: g/cm^3^	1.842
Grain size distribution	
<0.075 mm: %	63.3
0.075–0.1 mm: %	9.2
0.1–0.25 mm: %	20.2
0.25–0.5 mm: %	3.9
0.5–1 mm: %	3.4

**Table 2:** Mineral composition and relative content of binders.

Composition		CaO	SiO_2_	Al_2_O_3_	Fe_2_O_3_	MgO	Na_2_O	S	K_2_O	SO_3_	TiO_2_	Loss on Ignition ^b^
Content (%)	Cement	49.18	26.01	10.67	2.83	1.62	0.13	ND ^a^	0.95	3.76	0.51	3.54
	Lime	84.23	3.1	3.1	0.29	4.32	ND ^a^	0.13	ND ^a^	ND ^a^	ND ^a^	6.91
	Fly ash	5.73	39.65	21.42	9.17	3.68	2.03	ND ^a^	ND ^a^	ND ^a^	ND ^a^	18.32

^a^ ND: not detected; ^b^ Loss on ignition is the method described in [59] to measure organic matter content.

**Table 3:** Table of orthogonal design experiment scheme.

Test Type	Water Content: %	Freeze-Thaw Temperature: °C	Freeze-Thaw Cycles: d	Specimen ID ^b^
Long-term freeze-thaw	16.38 ^a^	−10 °C~20 °C	0, 3, 7, 14, 30, 90	C2.5S5F5Pb0.05, C5S2.5F2.5Pb0.05, C5S5Pb0.05 C2.5S5F5Pb0.5, C5S2.5F2.5Pb0.5, C5S5Pb0.5 C2.5S5F5Pb1, C5S2.5F2.5Pb1, C5S5Pb1
Microscopic test			0	Pb1
			0, 30, 90	C2.5S5F5Pb1, C5S2.5F2.5Pb1, C5S5Pb1

^a^ Water content is 120% of the optimal water content of the soil sample; ^b^ C, S, F, and Pb^2+^ stand for cement, lime, fly ash, and lead ions, respectively, and the number represents the mass percentage in dry soil. (e.g., C2.5S5F5Pb1 indicates that the mass percentages of cement, lime, fly ash, and lead to dry soil in the sample are 2.5%, 5%, 5%, and 1%, respectively).

## Supplementary Materials

The following are available online at https://www.mdpi.com/1660-4601/17/5/1798/s1.
