# Supplementary material for: Effects of Long-Term Repeated Freeze-Thaw Cycles on the Engineering Properties of Compound Solidified/Stabilized Pb-Contaminated Soil: Deterioration Characteristics and Mechanisms
Source: Int J Environ Res Public Health. 2020 Mar 10;17(5):1798. doi: 10.3390/ijerph17051798 (PMC7084238; doi:10.3390/ijerph17051798)
Supplement: Supplementary file 1 [file ijerph-17-01798-s001.pdf]

Table.S1 Result of nonparametric tests for the UCS of Pb-CSCSs

Binder ratios	Null hypothesis	Test	Significance	Decisions
C2.5S5F5	The distributions of the q_u of Pb-CSCSs with Pb^{2+} concentration 0.05%, 0.5% and 1% are the same.	Related-Samples Kendall's Coefficient of Concordance	0.115	Retain the null hypothesis
C5S2.5F2.5			0.030	Reject the null hypothesis
C5S5			0.223	Retain the null hypothesis

Note: the significance level is 0.05.

Table.S2 Pairwise comparison of the UCS of the C5S2.5F2.5 solidified Pb-contaminated soil

Sample1-Sample2	Test statistics	Standard error	Standard test statistics	Significance	Adjusted significance
0.05%-0.5%	−0.500	0.577	−0.866	0.386	1.000
0.05%-1%	1.500	0.577	2.598	0.009	0.028
0.5%-1%	1.000	0.577	1.732	0.083	0.250

Note: the significance level is 0.05.

Table.S3 Result of a nonparametric hypothesis test for the E_{50} of Pb-CSCSs

Binder ratios	Null hypothesis	Test	Significance	Decisions
C2.5S5F5	The distributions of the E_{50} of Pb-CSCSs with Pb^{2+} concentration 0.05%, 0.5% and 1% are the same.	Related-Samples Kendall's Coefficient of Concordance	0.607	Retain the null hypothesis
C5S2.5F2.5			0.607	Retain the null hypothesis
C5S5			0.002	Reject the null hypothesis

Note: the significance level is 0.05.

Table.S4 Pairwise comparison of the E_{50} of the C5S5 solidified Pb-contaminated soil

Sample1-Sample2	Test statistics	Standard error	Standard test statistics	Significance	Adjusted significance
0.05%-0.5%	1.000	0.577	1.732	0.083	0.250
0.05%-1%	2.000	0.577	3.464	0.001	0.002
0.5%-1%	1.000	0.577	1.732	0.083	0.250

Note: the significance level is 0.05.

Table.S5 Result of nonparametric hypothesis tests for the φ of Pb-CSCSs

Binder ratios	Null hypothesis	Test	Significance	Decisions
C2.5S5F5	The distributions of the φ of Pb-CSCSs with Pb^{2+} concentration 0.05%, 0.5% and 1% are the same.	Related-Samples Kendall's Coefficient of Concordance	0.016	Reject the null hypothesis
C5S2.5F2.5			0.115	Retain the null hypothesis
C5S5			0.115	Retain the null hypothesis

Note: the significance level is 0.05.

Table.S6 Pairwise comparison of the φ of the C2.55S5F5 solidified Pb-contaminated soil

Sample1-Sample2	Test statistics	Standard error	Standard test statistics	Significance	Adjusted significance
0.05%-0.5%	0.833	0.577	1.443	0.149	0.447
0.05%-1%	1.667	0.577	2.887	0.004	0.012
0.5%-1%	0.833	0.577	1.443	0.149	0.447

Note: the significance level is 0.05.

Table.S7 Result of nonparametric hypothesis tests for the *c* of Pb-CSCSs

Binder ratios	Null hypothesis	Test	Significance	Decisions
C2.5S5F5	The distributions of the *c* of Pb-CSCSs with Pb^{2+} concentration 0.05%, 0.5% and 1% are the same.	Related-Samples Kendall's Coefficient of Concordance	0.011	Reject the null hypothesis
C5S2.5F2.5			0.311	Retain the null hypothesis
C5S5			0.136	Retain the null hypothesis

Note: the significance level is 0.05.

Table.S8 Pairwise comparison of *c* of the C2.5S5F5 solidified Pb-contaminated soil

Sample1-Sample2	Test statistics	Standard error	Standard test statistics	Significance	Adjusted significance
0.05%-0.5%	0.000	0.577	0.000	1.000	1.000
0.05%-1%	−1.500	0.577	−2.598	0.009	0.028
0.5%-1%	−1.500	0.577	−2.598	0.009	0.028

Note: the significance level is 0.05.

Table.S9 Result of nonparametric hypothesis tests for the *k* of Pb-CSCSs

Binders ratios	Null hypothesis	Test	Significance	Decisions
C2.5S5F5	The distributions of the *k* of Pb-CSCSs with Pb^{2+} concentration 0.05%, 0.5% and 1% are the same.	Related-Samples Kendall's Coefficient of Concordance	0.002	Reject the null hypothesis
C5S2.5F2.5			0.607	Retain the null hypothesis
C5S5			0.009	Reject the null hypothesis

Note: the significance level is 0.05.

Table.S10 Pairwise comparison of *k* of the C2.55S5F5 (C5S5) solidified Pb-contaminated soil

Sample1-Sample2	Test statistics	Standard error	Standard test statistics	Significance	Adjusted significance
0.05%-0.5%	1.000 (0.333)	0.577 (0.577)	1.732 (0.577)	0.083 (0.564)	0.250 (1.000)
0.05%-1%	2.000 (1.667)	0.577 (0.577)	3.464 (2.887)	0.001 (0.004)	0.002 (0.012)
0.5%-1%	1.000 (1.333)	0.577 (0.577)	1.732 (2.309)	0.083 (0.021)	0.250 (0.063)

Note: the significance level is 0.05.

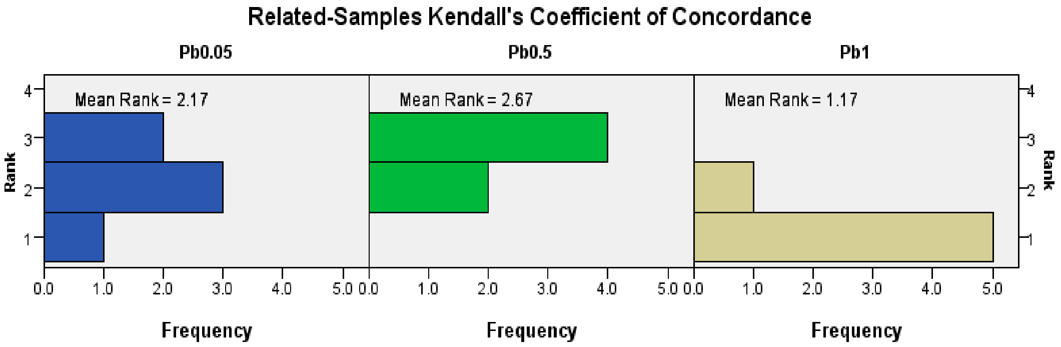

Fig.S1 The panel histogram of Kendall synergistic coefficient for related samples of the UCS of the C5S2.5F2.5 solidified Pb-contaminated soil

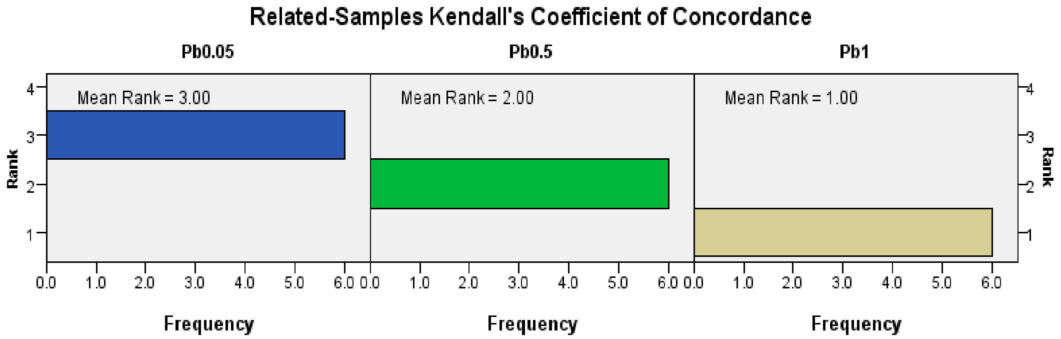

Fig.S2 Panel histogram of Kendal synergistic coefficient for related samples of the E50 of the C5S5 solidified Pb-contaminated soil

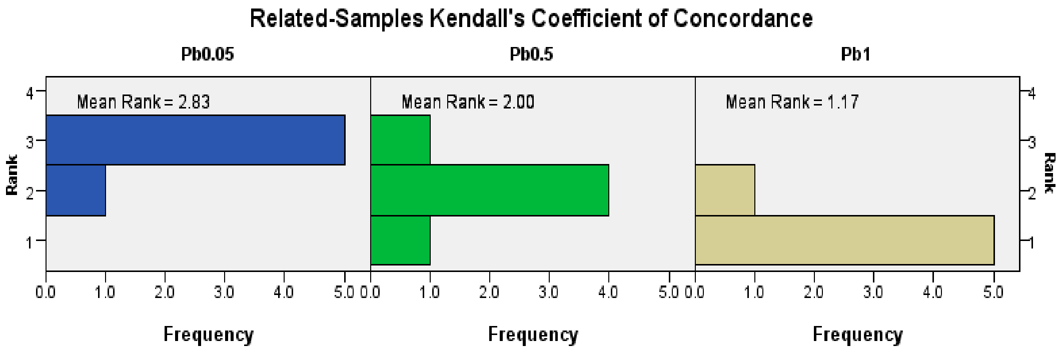

Fig.S3 Panel histogram of Kendall synergistic coefficient for related samples of the φ of the C2.55S5F5 solidified Pb-contaminated soil

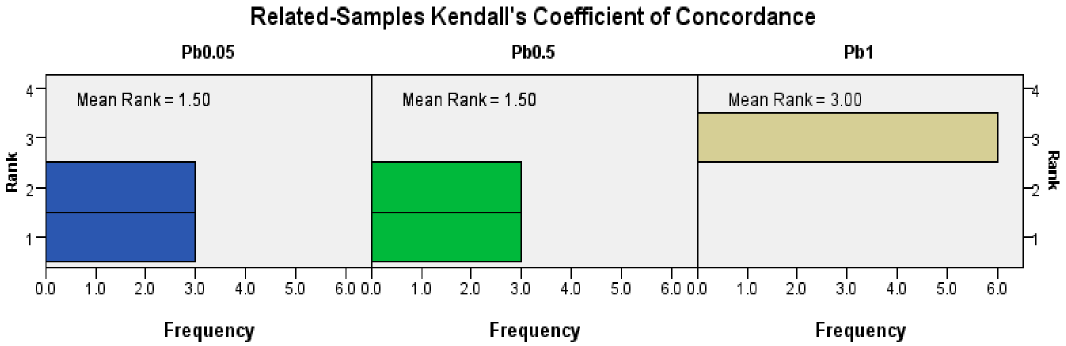

Fig.S4 Panel histogram of Kendall synergistic coefficient for related samples of the c of the C2.5S5F5 solidified Pb-contaminated soil

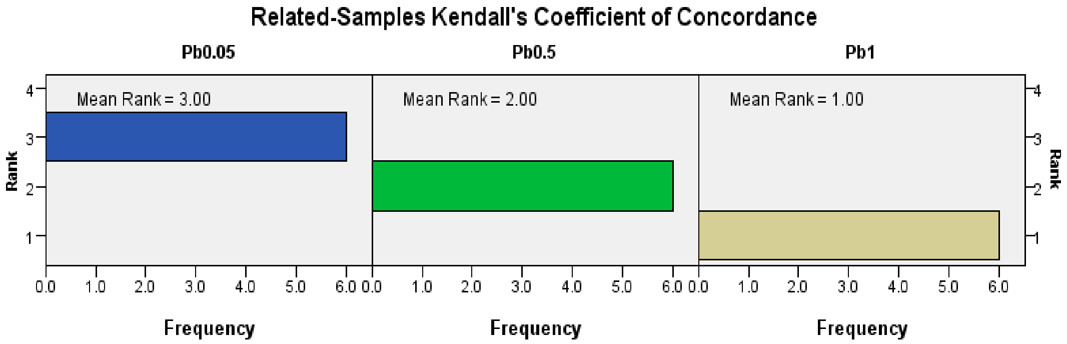

(a)C2.5S5F5

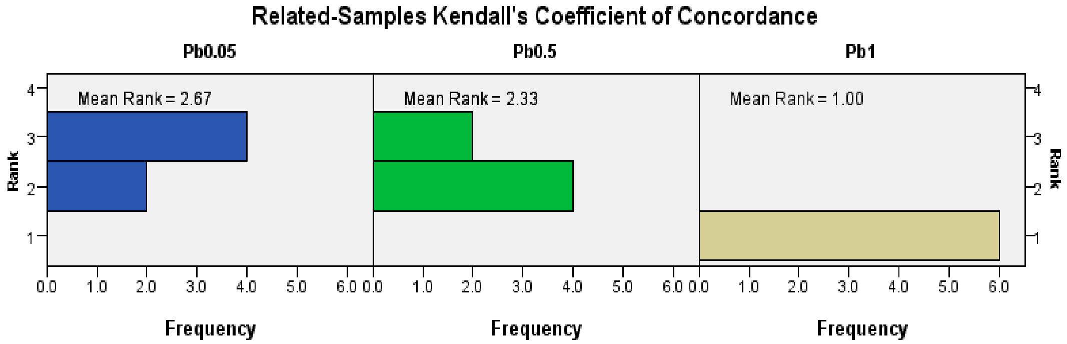

(b)C5S5

Fig.S5 Panel histogram of Kendall synergistic coefficient for related samples of *k* of the C2.5S5F5 and the C5S5 solidified Pb-contaminated soil
